# Vermicompost Improves Tomato Yield and Quality and the Biochemical Properties of Soils with Different Tomato Planting History in a Greenhouse Study

**DOI:** 10.3389/fpls.2017.01978

**Published:** 2017-11-21

**Authors:** Xin-Xin Wang, Fengyan Zhao, Guoxian Zhang, Yongyong Zhang, Lijuan Yang

**Affiliations:** Plant Nutrition and Fertilizer, Land and Environmental College, Shenyang Agricultural University, Shenyang, China

**Keywords:** tomato, planting years, soil biochemical properties, vermicompost, greenhouse study

## Abstract

A greenhouse pot test was conducted to study the impacts of replacing mineral fertilizer with organic fertilizers for one full growing period on soil fertility, tomato yield and quality using soils with different tomato planting history. Four types of fertilization regimes were compared: (1) conventional fertilizer with urea, (2) chicken manure compost, (3) vermicompost, and (4) no fertilizer. The effects on plant growth, yield and fruit quality and soil properties (including microbial biomass carbon and nitrogen, ${\text{NH}}_{4}^{+}$-N, ${\text{NO}}_{3}^{-}$-N, soil water-soluble organic carbon, soil pH and electrical conductivity) were investigated in samples collected from the experimental soils at different tomato growth stages. The main results showed that: (1) vermicompost and chicken manure compost more effectively promoted plant growth, including stem diameter and plant height compared with other fertilizer treatments, in all three types of soil; (2) vermicompost improved fruit quality in each type of soil, and increased the sugar/acid ratio, and decreased nitrate concentration in fresh fruit compared with the CK treatment; (3) vermicompost led to greater improvements in fruit yield (74%), vitamin C (47%), and soluble sugar (71%) in soils with no tomato planting history compared with those in soils with long tomato planting history; and (4) vermicompost led to greater improvements in soil quality than chicken manure compost, including higher pH (averaged 7.37 vs. averaged 7.23) and lower soil electrical conductivity (averaged 204.1 vs. averaged 234.6 μS/cm) at the end of experiment in each type of soil. We conclude that vermicompost can be recommended as a fertilizer to improve tomato fruit quality and yield and soil quality, particularly for soils with no tomato planting history.

## Introduction

Intensive agricultural production using inorganic fertilizers has led to increased yield, albeit at the expense of poor product quality, particularly under protected cultivation.

Tomato (*Lycopersicon esculentum*) is one of the most widely grown vegetables in Liaoning Province, where the tomato-growing area encompassed more than 85,000 hectares in 2014 (Zhao et al., 2015). However, determining how to improve tomato quality without reducing fruit yield remains an urgent unsolved problem. The use of organic farming with organic amendments as nutrient inputs to the soil is currently increasing, and organic farming is becoming an alternative agricultural practice to sustain economical vegetable production with minimal environmental pollution and higher fruit quality.

It is widely acknowledged that using composts and vermicomposts as amendments, rather than industrialized fertilizer and raw manure, could improve soil nutrients and promote soil health (Jack and Thies, 2006). Manure compost has been widely applied as it is highly accessible at low price (Hepperly et al., 2009; Ramirez-Guerrero and Meza-Figueroa, 2014), and greatly improved most of the characteristics of crop plants compared with mineral fertilizer (Da Silva et al., 2011). Vermicomposts are finely divided, peat-like materials produced through a non-thermophilic process involving the biodegradation and stabilization of organic materials through interactions between earthworms and microorganisms (Edwards and Burrows, 1988). Vermicomposts are characterized by high porosity, aeration, drainage, water-holding capacity and microbial activity. Many studies have demonstrated positive effects of vermicompost on a wide range of crops, including cereals and legumes, ornamental, and flowering plants (Chan and Griffiths, 1988), vegetables (Edwards and Burrows, 1988; Subler et al., 1998; Atiyeh et al., 2000), and field crops (Mba, 1996). Application of compost and vermicompost can also increase soil organic carbon, nitrates, phosphates, exchangeable calcium and some other nutrients for plants (Orozco et al., 1996; Garcia-Gil et al., 2000; Bulluck et al., 2002; Jindo et al., 2016). Most of these investigations have confirmed that manure compost and vermicompost usually has significant beneficial effects on plant growth. However, there have been very few experimental investigations exploring effects of vermicompost and manure compost applications on tomato.

Tomato-producing systems include many tomato-growing solar greenhouses with different tomato planting history. The soils with long tomato planting history cause soil degradation (such as soil acidification), soil nutrient enrichment (such as Olsen-P, total nitrogen, and available potassium; Fu et al., 2017), or decreased soil microbial diversity compared to the soil with short tomato planting history (Zhang et al., 2015). Thus, additional investigation is needed to determine whether applying vermicompost and manure compost to soils with different tomato planting history can provide the same benefits for tomato plants in terms of yield and quality.

Our main objective was to investigate the effects of vermicompost applications on tomato growth, yield and fruit quality grown in soils with different tomato planting history. The hypothesis of this study was that the effects of vermicompost on tomato yield and quality, as well as soil quality, would differ among soils with different tomato planting history due to alterations in soil traits caused. To investigate this hypothesis, a pot experiment was conducted in a greenhouse using four fertilizer treatments, including vermicompost, compost, urea, and no fertilizer, on soils with 0, 5, and 20 years of tomato planting history.

## Materials and methods

### Experimental site and materials

The original soil for the experiment was collected from greenhouse tomato plants in the Guanghui township of Yuhong District, southwest Shenyang City, Liaoning Province. The soils had different years of continuous cropping: 0 (from a corn field adjacent to the tomato-planting greenhouses), 5, and 20 years.

A pot experiment was conducted from March to June of 2016 at the Greenhouse Base (123°57′ E, 41°83′ N) of Shenyang Agricultural University in Shenyang City, Liaoning Province. Polyethylene plastic pots with a diameter of 30 cm and a height of 28 cm were used. Each pot was filled with 15 kg of air-dried soil that had been passed through 1 cm sieve. The basic physical and chemical properties of the soil are shown in Table 1. The vermicompost used in the study was obtained by adding earthworms (*Eisenia fetida*) to semi-decomposed cow manure; before use, the vermicompost was passed through a 2 mm sieve to remove the earthworms. Chicken manure compost was purchased from Ruiyuande Biotechnology Co., Ltd. (Shenyang, Liaoning Province, China). The nutrient content of the fertilizer is shown in Table 2. The tested tomato variety, “Gold Crown No. 9,” was grown until all the fruits were harvested.

**Table 1:** Basic characters of cultivated soil used in experiment.

**Cropping years**	**pH**	**Electrical conductivity**	**Available N**	**Available P**	**Available K**	**Organic matter**
		**μS/cm**	**mg/kg**	**mg/kg**	**mg/kg**	**g/kg**
0	8.14	130	101.79	28.15	152.73	20.75
5	7.85	250	135.93	158.56	231.76	31.46
20	6.17	490	206.94	374.03	564.57	46.63
**Cropping years**	**Total C**	**Total N**	**C/N**	**Available Ca**	**Available Mg**	
	**g/kg**	**g/kg**		**g/kg**	**g/kg**	
0	12.76	1.32	11.23	1.42	0.08	
5	15.72	1.84	10.18	1.11	0.1	
20	25.4	3.07	8.93	1.03	0.1	

**Table 2:** Nutrient content in various manures or fertilizers.

**Item**	**Vermicompost**	**Compost**	**Urea**	**Calcium superphosphate**	**Potassium sulfate**
pH	6.52	6.34	7.02	5.90	6.80
EC (μS/cm)	2,190.00	17,850.00	–	–	–
OM (g/kg)	102.44	430.48	–	–	–
Total N (g/kg)	7.68	29.97	460.00	–	–
Total P_2_O_5_ (g/kg)	19.09	30.13	–	120.00	–
Total K_2_O (g/kg)	4.13	7.20	–	–	500.00
Total Ca (g/kg)	25.10	7.22	–	0.07	–
Total Mg (g/kg)	2.92	2.03	0.005	1.40	0.03

### Pot experiment design

The experiment had a randomized complete full factorial block design with two factors. Four fertilization treatments were applied to three soils with different years of continuous cropping (0, 5, and 20 years). Each treatment was repeated five times with a complete random arrangement within each block. The four fertilization treatments were CK (control: no fertilizer), urea (chemical fertilizer: 0.4 g N, 0.25 g P_2_O_5_, and 0.4 g K_2_O per kg soil that is equal to 900 kg N, 560 kg P_2_O_5_, and 900 kg K_2_O per hectare), compost (chicken manure compost), and vermicompost. A total of 8.30 g of chicken manure compost per kg soil (about 19 t chicken manure compost per hectare) and 13.10 g of vermicompost per kg soil (about 30 t vermicompost per hectare) was added to the compost and vermicompost treatments, respectively (Table 2). The added amounts of compost and vermicompost were calculated according to their P concentrations due to its relatively higher content. To ensure that the concentrations of added N, P, and K were equal among the urea, compost and vermicompost treatments, additional chemical N and K were added to the latter two treatments to achieve the same N, P, and K contents as the urea treatment. Chemical N, P, and K were applied as urea, superphosphate and potassium sulfate, respectively. All the fertilizers were applied as basal fertilizer in a one-time application.

On March 12, 2016 (~35 days after seeding), when the tomato seedlings had grown to the 3- to 4-leaf stage, seedlings of a consistent size were transplanted into each pot. The plants were watered to 100% field water-holding capacity using tap water. After this watering, all the treatments received the same amount of irrigation. Soil moisture was maintained at 18–20% (w/w, i.e., 70% of field water-holding capacity) during the experiment, as determined gravimetrically, with the addition of tap water when necessary.

The pots within a block were arranged randomly in the greenhouse, with the positions re-randomized every week. The greenhouse temperature ranged from 15 to 35°C. Natural light was supplied with no supplementary light.

### Soil characteristics

During the entire growth period of the tomato plants, soil samples were collected in duplicate from each pot on March 27 (seedling stage), April 26 (flowering stage), May 26 (fruiting stage), and June 25 (harvesting stage; 105 days). Part of the fresh soil was passed through a 2 mm sieve and stored at 4°C for other measurements, and the remainder of the soil was air-dried and passed through a 0.9 and a 0.15 mm sieve for the determination of physical and chemical properties.

Soil physical and chemical properties were determined as previously described (Bao, 2001). The soil pH (soil: water, 1:5) and electrical conductivity (EC) values (soil: water, 1:5) were measured using a Thunder Magnetic SJ-3F pH Meter (INESA, Shanghai, China) and a DDS-307 conductivity meter (INESA, Shanghai, China), respectively. The soil total carbon (TC), total nitrogen (TN) and C:N ratio were determined using an elemental analyzer (Elementar III, Germany).

The soil microbial biomass carbon (MBC) and microbial biomass nitrogen (MBN) were determined according to Brookes et al. (1985) and Vance et al. (1987) for chloroform fumigation and potassium sulfate extraction. Each fresh soil sample (equivalent to ~20 g of air-dried soil) was fumigated for 24 h at 25°C in the dark in a vacuum desiccator with ethanol-free chloroform. At the same time, each sample was fumigated without ethanol-free chloroform under the same conditions as a control. All the soil samples were extracted with 0.5 M K_2_SO_4_ (1/4 w/v). The MBC and MBN contents in the extract were measured with a Multi N/C^®;^ 3100 analyzer (Analytik Jena, Germany) and were calculated using calibration factors of 0.45 (Vance et al., 1987) and 0.54 (Brookes et al., 1985), respectively. Water-soluble organic carbon (WSOC) was determined according to Chantigny et al. (2010). Each soil sample (~5 g of air-dried soil) was added to 45 mL of ultrapure water, shaken at 180 r/min for 1 h, and centrifuged at 4,000 r/min for 15 min. The supernatant was then passed through a 0.45 μm filter, and the filtrate was stored at 4°C until use. The WSOC contents in the filtrate were quantified using a Multi N/C® 3100 analyzer (Analytik Jena, Germany). Nitrate nitrogen (${\text{NO}}_{3}^{-}$-N) and ammonium nitrogen (${\text{NH}}_{4}^{+}$-N) were extracted from the soil using 0.01 mol/L CaCl_2_ (10:1 v/w), and the ${\text{NO}}_{3}^{-}$-N and ${\text{NH}}_{4}^{+}$-N contents in the extract were measured using an AA3 Continuous-Flow Analyzer (Bran+Luebbe, Germany).

### Plant parameters

Each tomato fruit was weighed, and the total yield per plant was calculated. The plant height, stem diameter and leaf chlorophyll content (SPAD) were measured at 15 days (March 27), 45 days (April 26), 75 days (May 26), and 105 days (June 25) after transplanting. The chlorophyll content of the leaves at the same part of the second branch of the plant was measured using the CCM-200 Plus Chlorophyll Analyzer (OPTI-SCIENCES, USA), and the average of three measurements was calculated. The chlorophyll content measurement was based on the difference in the light absorption rate at 653 and 931 nm.

Fruit quality was determined in tomatoes of similar color when the second ear fruit reached at 75–85% maturity (Li, 2000; Bao, 2001). The vitamin C content was determined using 2, 6-dichlorophenolindophenol and was expressed in milligrams of vitamin C per 100 g of fresh sample (mg/100 g). The total acidity was determined using the neutralization titration method and was expressed as a percentage (%). The soluble sugar contents were determined using the cyanide iodine method and expressed as a percentage (%). The nitrate contents were measured using salicylic acid colorimetry and expressed in mg/kg.

### Data analysis

The data was presented as the arithmetic mean values with standard errors. One-way ANOVA was performed to compare the effects of different fertilization treatments on tomato yield and fruit quality. Two-ways ANOVA was done on stem diameter, plant height, SPAD, ${\text{NH}}_{4}^{+}$-N, ${\text{NO}}_{3}^{-}$-N, MBC, MBN, TC, TN, WSOC, EC, and pH on different soil. Differences at the 5% significance level were compared using Tukey's Honestly Significant Difference (HSD) test. ANOVA was performed on stem diameter, plant height and SPAD values, and the means were compared using Tukey's HSD test at the 5% significance level. ANOVA was used to analyze the interaction between fertilizer treatments and sampling days. A simple Pearson's correlation analysis was performed to correlate ${\text{NH}}_{4}^{+}$-N, ${\text{NO}}_{3}^{-}$N, and water-soluble carbon contents with plant growth parameters and fruit quality. The statistical analyses were performed with SPSS software, version 20.0 (IBM Corp., Armonk, NY, USA).

## Results

### Plant growth and SPAD

**Figure 1:**
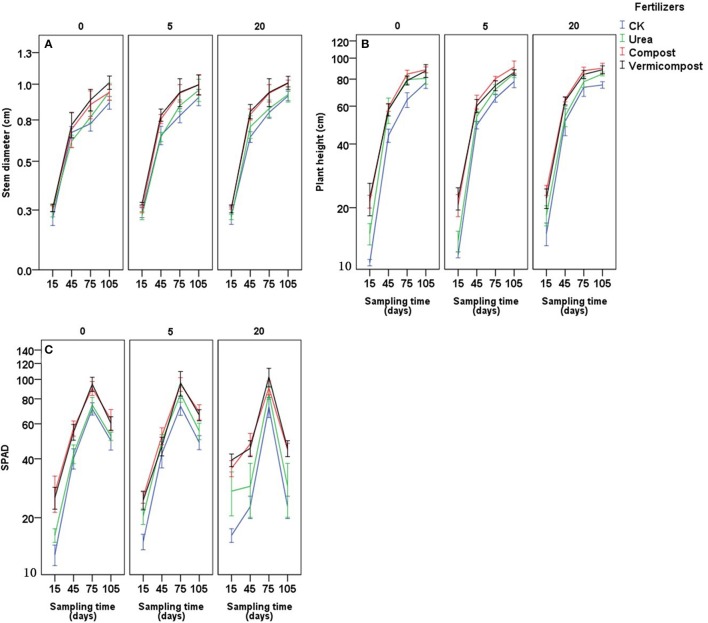
Effects of fertilizers and sampling time (days after transplanting) on stem diameter **(A)** plant height **(B)** and SPAD **(C)** on soils with 0, 5, and 20 years of tomato planting history.

The stem diameter, plant height and SPAD values were influenced by the fertilizer treatment in each type of soil (Table 3 and Figure 1; *P* < 0.001). There was no interaction for these three parameters between sampling days and fertilizer treatments (Table 3), indicating that fertilizer treatment had a consistent effect on these parameters. The stem diameter differed significantly among the treatments: in soil with 0 cropping years, the effect ranking was vermicompost ≥ compost ≥ urea ≥ CK; after 5 or 20 continuous cropping years, the effect was vermicompost ≥ compost > urea ≥ CK. The plant height also differed significantly: in soil with 0 cropping years, the effect ranking was vermicompost ≥ compost ≥ urea > CK; after 5 or 20 continuous cropping years, the effect was vermicompost ≥ compost > urea > CK. Moreover, SPAD values differed significantly among the treatments: in soil with 0 cropping years, the effect ranking was vermicompost ≥ compost > urea ≥ CK; after 5 or 20 continuous cropping years, the effect was vermicompost ≥ compost > urea > CK (Table 3).

**Table 3:** Results of analysis of variance (*P*-values) for stem diameter, plant height, and SPAD treated with different fertilizers on soils at four sampling times after transplanting with different tomato planting history.

**Sources**	**0 Continuous cropping years**			**5 Continuous cropping years**			**20 Continuous cropping years**		
	**Stem diameter**	**Plant height**	**SPAD**	**Stem diameter**	**Plant height**	**SPAD**	**Stem diameter**	**Plant height**	**SPAD**
Fertilizers(F)	9.440^***^	39.993^***^	40.367^***^	14.516^***^	42.870^***^	22.091^***^	19.994^***^	34.560^***^	54.275^***^
Sampling(S)	436.436^***^	863.129^***^	386.948^***^	552.872^***^	1, 210.347^***^	338.368^***^	695.772^***^	887.172^***^	318.091^***^
F*S	1.389	1.917	1.019	1.332	1.079	1.806	1.180	0.794	1.011

*F-values are shown*.

*** *P < 0.001; indicating significance*.

### Yield and fruit quality

**Figure 2:**
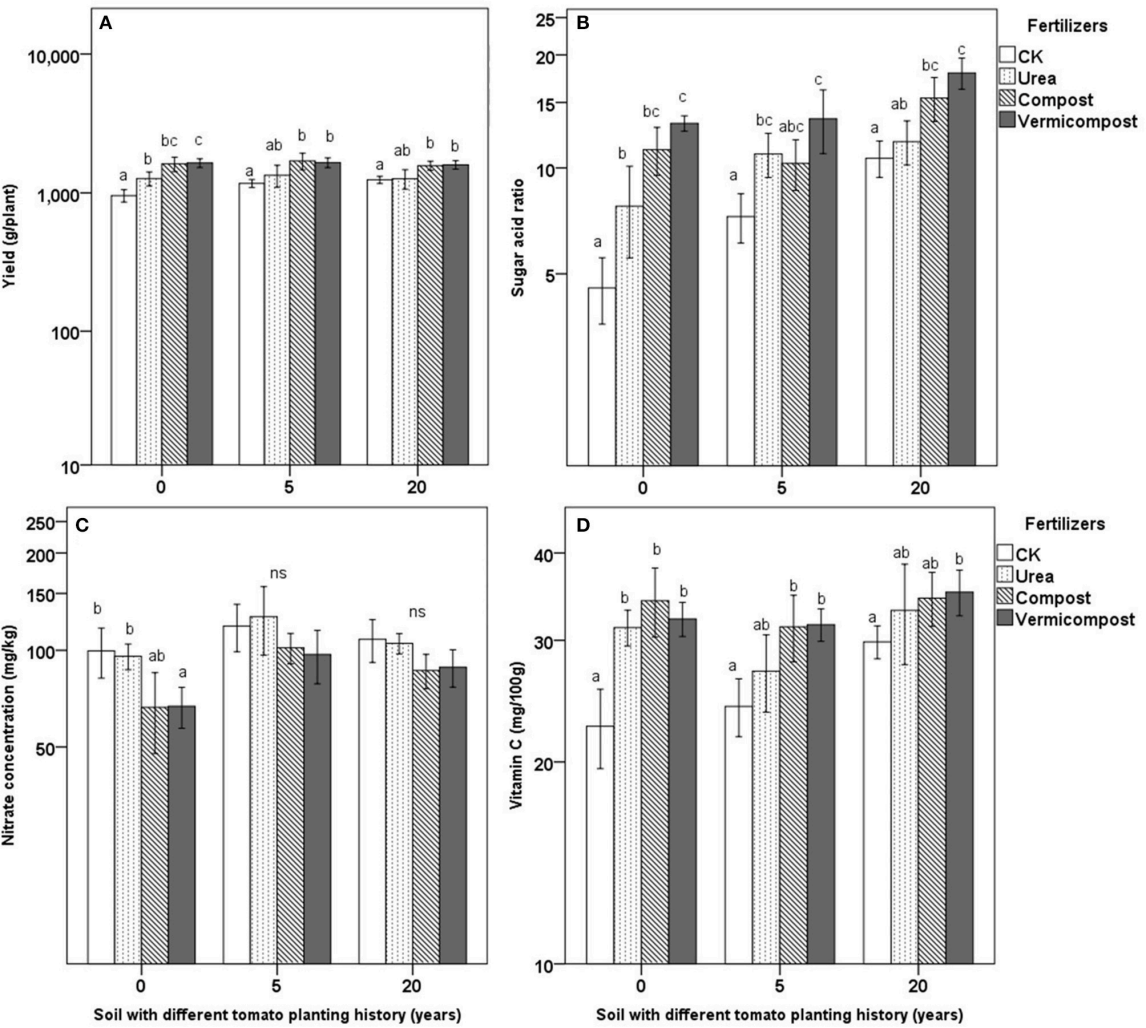
Effect of fertilizers on tomato yield **(A)**, sugar acid ratio **(B)**, nitrate concentration **(C)** and vitamin C **(D)** on soils with 0, 5, and 20 continuous tomato planting years. Means in the same soil followed by different letters denote significant differences according to Tukey test (*P* < 0.05).

There were significant effects of fertilizers on tomato yield (Figure 2A). In soil with 0 cropping years, the treatment of vermicompost had the highest tomato yield (1,642 g/plant; followed by that of the compost treatment, 1,616 g/plant), which was significantly higher than that of the urea treatment (1,268 g/plant) and the CK treatment (956 g/plant; Figure 2A); after 5 cropping years, the tomato yield of the compost treatment and the vermicompost treatment had the similar value (1,697 and 1,654 g/plant, respectively), followed by that of the urea treatment and CK treatment (1,340 and 1,169 g/plant, respectively; Figure 2A); the tread of tomato yield in different fertilizer treatments kept the same after 20 cropping years (Figure 2A).

Different fertilizers had significant effects on tomato quality, including the sugar/acid ratio, nitrate concentration and vitamin C concentration in fruit (Figures 2B–D). Sugar/acid ratios in the treatment of vermicompost had the highest value in the each type of soil, significantly higher than that in the treatment of urea (Figure 2B). In each type of soil, the treatment of compost and vermicompost had the relatively lower the fruit nitrate concentration compared with that in the treatment of CK and urea. Particularly in the soil with 0 years tomato plant history, nitrate concentration of fruit in the vermicompost treatment was significantly lower than that in the CK treatment (67 vs. 100 mg/kg; Figure 2C). Compared with the CK, all the three fertilizer application improved vitamin C content, but only the vermicompost significantly improved vitamin C content in the each type of soil (Figure 2D).

### Soil nitrogen

**Figure 3:**
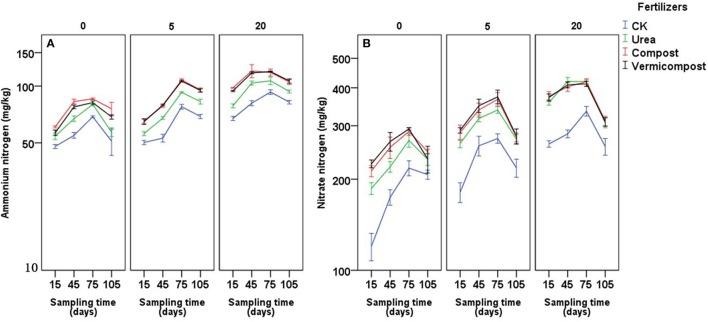
Effects of fertilizers and sampling time (days after transplanting) on ammonium-N **(A)** and nitrate-N **(B)** of soils on soils with 0, 5, and 20 years of tomato planting history.

Different fertilizers, sampling days and their interaction had significant effects on soil ${\text{NH}}_{4}^{+}$-N and ${\text{NO}}_{3}^{-}$-N (Table 4). An examination of the data presented in Figures 3A,**B** shows that the amounts of soil ${\text{NH}}_{4}^{+}$-N, and ${\text{NO}}_{3}^{-}$-N were greater 15 days after treatment of the soil with fertilizers compared with those in the CK treatment. In most cases, the soil ${\text{NH}}_{4}^{+}$-N content followed the following order: compost ≥ vermicompost > urea > CK; the soil ${\text{NO}}_{3}^{-}$-N content followed the order: vermicompost ≥compost > urea > CK.

**Table 4:** Results of analysis of variance (*P* values) for ${\text{NH}}_{4}^{+}$-N, ${\text{NO}}_{3}^{-}$-N, microbial biomass carbon (MBC), microbial biomass nitrogen (MBN), total nitrogen (TN), water soluble organic carbon, electric conductivity, and pH at four sampling days on soils with different tomato planting history.

**Cropping years**	**Sources**	**${\text{NH}}_{4}^{+}$-N**	**${\text{NO}}_{3}^{-}$-N**	**MBC**	**MBN**	**TC**	**TN**	**WSOC**	**EC**	**pH**
0	Fertilizers(F)	135.299^***^	121.301^***^	58.099^***^	32.251^***^	34.934^***^	114.17^***^	134.657^***^	1, 105.797^***^	548.461^***^
	Sampling(S)	182.102^***^	112.194^***^	6.060^**^	10.818^***^	7.165^***^	31.96^***^	43.622^***^	1, 969.006^***^	290.237^***^
	F^*^S	5.927^***^	5.636^***^	11.260^***^	2.809^**^	1.832	10.747^***^	12.149^***^	246.102^***^	38.758^***^
5	Fertilizers(F)	675.067^***^	136.207^***^	118.156^***^	31.104^***^	34.745^***^	149.45^***^	52.894^***^	944.463^***^	2, 878.032^***^
	Sampling(S)	1379.033^***^	134.909^***^	8.094^***^	7.569^***^	9.53^**^	112.225^***^	44.765^***^	2, 643.535^***^	1, 887.606^***^
	F^*^S	11.666^***^	2.205^*^	13.702^***^	5.312^***^	3.027^**^	9.017^***^	5.904^***^	250.43^***^	310.74^***^
20	Fertilizers(F)	254.566^***^	291.702^***^	76.802^***^	55.714^***^	54.145^***^	33.318^***^	379.351^***^	1, 080.389^***^	2, 815.771^***^
	Sampling(S)	162.367^***^	269.2^***^	12.636^***^	44.121^***^	4.719^**^	17.854^***^	114.476^***^	2, 393.522^***^	4, 032.17^***^
	F^*^S	3.722^**^	10.369^***^	9.245^***^	4.29^***^	4.644^**^	1.336	91.338^***^	218.264^***^	233.511^***^

*F-values are shown*.

* P < 0.05;

** P < 0.01;

*** *P < 0.001; indicating significance*.

### Soil EC and pH

In all three types of soil, both soil EC and pH were influenced by sampling time, fertilizer treatment, and the interaction between them (Table 4 and **Figure 5**). Overall, compared with CK treatment, the other fertilizer treatments (particularly compost and urea) largely promoted soil EC at the beginning of the experiment. Over time, soil EC decreased to a much lower level than at the beginning (**Figure 5A**). At the final sampling period, after the entire growth stage, soil EC exhibited the following order: compost > vermicompost > urea > CK, regardless of years of continuous cropping.

Soil pH was highest in the CK group at almost every stage and cropping span, whereas urea treatment produced the lowest soil pH at almost every stage in soil with 0 and 20 years cropping history (**Figure 5B**). In soil with 0 cropping years, treatment with urea, compost and vermicompost produced average (from four sampling times) reductions in soil pH of −0.45, −0.27, and −0.26, respectively, compared with CK treatment; after 5 cropping years, the reduction was −0.46, −0.42, and −0.42, respectively, compared with CK treatment; and after 20 cropping years, the reduction was −0.59, −0.23, and −0.14, respectively, compared with CK. At the final growth stage, soil pH exhibited the following orders: urea ≤ compost < vermicompost < CK after 0 cropping years; compost ≤ vermicompost < urea < CK after 5 cropping years; and urea < compost < CK < vermicompost after 20 cropping years.

### Water-soluble organic carbon in soil

**Figure 4:**
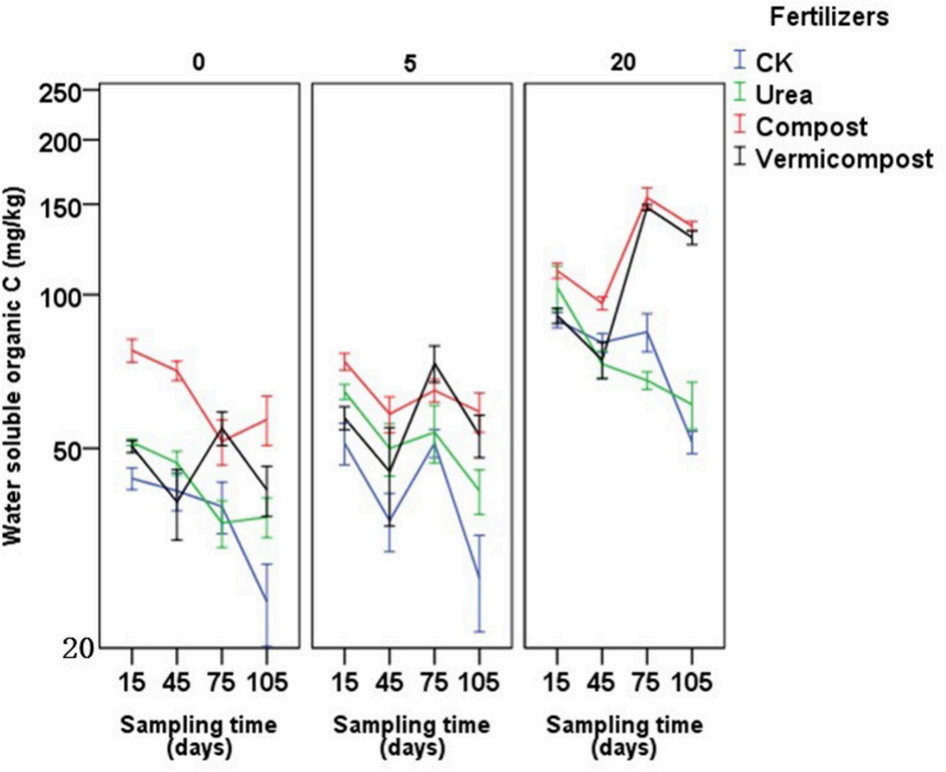
Effects of fertilizers and sampling time (days after transplanting) on water soluble organic carbon of soils on soils with 0, 5, and 20 years of tomato planting history.

Different fertilizer treatments, sampling days and their interaction had significant effects on the soil water-soluble organic carbon content at all four growing periods (Table 4). In most of cases, the compost treatment had the highest the soil water-soluble organic carbon content (Figure 4). In the each types of soil, averaged cross sampling days, the soil water-soluble organic carbon content followed the following order: compost > vermicompost > urea > CK.

**Figure 5:**
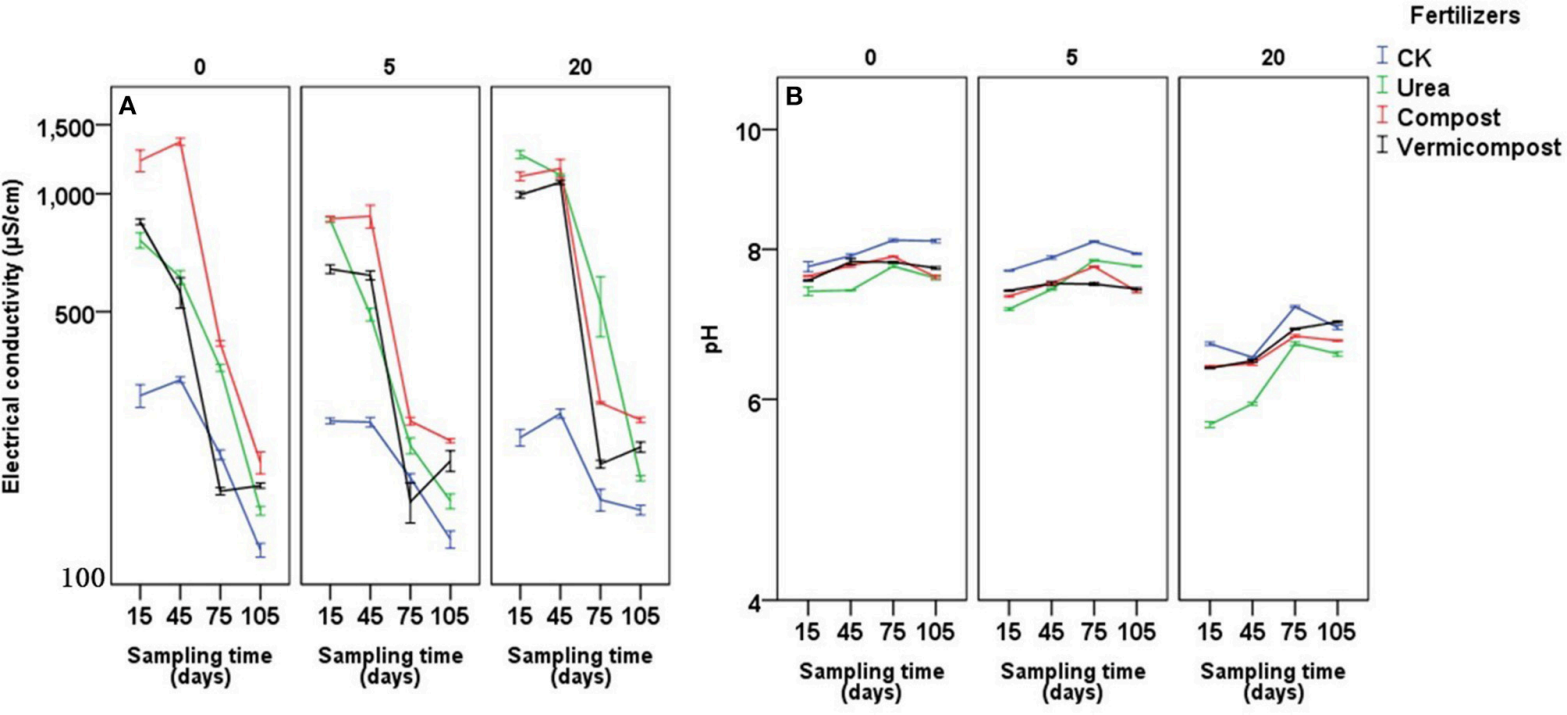
Effects of fertilizers and sampling time (days after transplanting) on electrical conductivity **(A)** and pH **(B)** of soils on soils with 0, 5, and 20 years of tomato planting history.

### Effects of vermicompost on fruit yield and fruit quality among soils with different tomato planting history

**Table 6** shows differences in the effects of vermicompost on fruit yield and fruit quality in soils with different years of continuous cropping. The increases (%) in the yield, sugar/acid ratio, vitamin C and soluble sugar of fruit were the highest in soil with 0 cropping years. These differences were significantly higher than those after 20 cropping years while the differences after 5 cropping years were between 0 and 20 years. Furthermore, the decrease (%) in nitrate and organic acids in soil with 0 cropping years were the highest among the three soil treatments.

### Correlations between soil parameters and yield and quality

A correlation matrix among the different yields, quality parameters, and soil parameters found in the present study is presented in Table 5. The correlation matrix showed that tomato yield had a significant and positive correlation with nitrogen nutrient availability under all the fertilizer treatments (${\text{NH}}_{4}^{+}$-N: *r* = 0.459, *P* < 0.01; ${\text{NO}}_{3}^{-}$-N: *r* = 0.447, *P* < 0.01). Vitamin C, soluble sugar, acid content and sugar/acid ratio were significantly correlated with the water-soluble organic carbon, ${\text{NH}}_{4}^{+}$-N and ${\text{NO}}_{3}^{-}$-N in the soil.

**Table 5:** Correlations tomato yield and quality with soil quality indicators of the different fertilizer treatments.

	**Average WSOC**	**Average soil ${\text{NH}}_{4}^{+}$-N**	**Average soil ${\text{NO}}_{3}^{-}$-N**
Yield	0.246	0.459^**^	0.447^**^
Vitamin C	0.444^**^	0.591^**^	0.518^**^
Nitrate concentration	−0.166	−0.132	0.034
Sugar/acid ratio	0.340^**^	0.770^**^	0.739^**^
Soluble sugar	0.317*	0.727^**^	0.703^**^
Organic acids	−0.405*	−0.625^**^	−0.651^**^

*r-value was shown*.

* P < 0.05;

** *P < 0.01 Indicating significance (Person coefficient, P < 0.05)*.

## Discussion

### Effects of vermicompost on tomato growth and yield in soils with different continuous growth history

Higher plant height and thicker stem diameter were found after the treatments with vermicompost, which is consistent with previous research showing that crop plants had increased height after vermicompost was applied (Kmet'ova and Kovacik, 2013). This result could be due to the higher nitrogen content in soil caused by applying vermicompost in this experiment (Figure 3). Singh and Varshney (2013) found that soil ${\text{NH}}_{4}^{+}$-N and ${\text{NO}}_{3}^{-}$-N were immediately improved after applying vermicompost. Vermicompost can also enhance the growth of nitrogen-fixing microorganisms in the rhizosphere, which enhances N availability by making biologically fixed N available through the intimate mixing of ingested particles with soil (Mackay et al., 1982). Later, Arancon et al. (2003) indicated the improvements in crop growth and increase in fruit yields could also be due to partially to large increase in soil microbial biomass after application of vermicompost, leading to the more hormones or humate content in the vermicompost treatment. We did find increased microbial biomass carbon and nitrogen in the vermicompost (Figure S1). Thus, a high nutrient or hormones status of soil with vermicompost may improve the speed of tomato growth. Moreover, higher SPAD values were found in plants treated with vermicompost and compost than those in CK and urea treatment (Figure 1C). The correlation between the leaf chlorophyll content index and plant nitrogen content has been demonstrated to be useful for estimating plant nitrogen status (Li et al., 2009; Cabangon et al., 2011; Yuan et al., 2016). SPAD values accurately reflect the quantity of mineral N required by plants (Van Den Berg and Perkins, 2004). SPAD values could also demonstrate that vermicompost and compost improve the soil N status and thus are taken up by plants. Manh and Wang (2014) found that vermicompost had strongly positive effects on the seedling quality and growth of muskmelon (*Cucumis melo* L.). Singh and Chauhan (2009), applied the vermicompost to the French bean (*Phaseolus vulgaris*), finding that the vermicompost promoted the germination of seeds, the height of the plant, the number of leaves of each plant, length of leaves and width of leaves of each plant and the yield. Thus, applying organic fertilizer produced an improvement in plants even at the beginning of the growth cycle. The data analysis confirmed that the final tomato yield was correlated with soil N status (Table 5). Lastly, it was found that applying Ca fertilizer could improve about 10% of yield (Qin et al., 2008), that may be due to nutrient of Ca plays a key role in fruit growth and development (Kadir, 2005). In our experiment, both vermicompost and compost contains substantial Ca content (Table 1), which could be a reason for relatively high yield in these treatments.

The available nutrient content in soil increased with the increasing continues tomato-planting years (Table 1 and Figure 3); however, the increased available N content did not lead to an increased tomato yield among soils with different years (Figure 2). We speculated two reasons which could explain this phenomenon. Firstly, the available Ca content in soils decreased with the increasing continues tomato-planting years (Table 1). Lack of available Ca may be the limiting factor for the yield in the soil with 5 or 20 years of tomato planting history (Kadir, 2005). Secondly, the long-term cultivation of a single crop may lead to the accumulation of harmful microbes, and the diversity and number of beneficial bacteria may decrease with the increasing continuous cropping years (Zheng et al., 2005). The diversity of the soil microbial community gradually decreased with the increase of the continuous monoculture tomato in a solar greenhouse (Fu et al., 2017). Despite that the obvious symptom was not observed in our experiment, it was still possible that the accumulative harmful microbes in the long tomato-planting history could inhibit tomato' growth and yield. The test on the microbial diversity, particularly the richness and abundance of pathogens, will be needed in the future research.

### Effects of vermicompost on tomato quality in soils with different continuous growth history

The results of this study showed that tomato quality was significantly influenced by fertilizer treatments, regardless of the number of years of continuous cropping. The sugar/acid ratio and nitrate content were higher and lower, respectively, in the vermicompost-treated plants than in plants with the other fertilizer treatments, in consistent with the results of Yang et al. (2015). For cucumber, the addition vermicompost or vermicompost with organic-inorganic mixed fertilizers significantly improved the overall quality of cucumber, including the ratio of sugar to organic acid, vitamin C and soluble protein in greenhouse compared with addition of pure inorganic fertilizer and pure chick manure compost (Zhao et al., 2010). Vermicompost is rich in potassium (Hanc and Vasak, 2015; Mondal et al., 2015), and Çolpan et al. (2013) found that potassium improved the yield and fruit quality of tomato. Thus, the tomato quality improvement observed in the present work could be due to the increased potassium. Moreover, water-soluble organic carbon was enhanced by the addition of organic fertilizer (Figure 4). Qualities such as the vitamin C and soluble sugar contents in fruit were significantly positively correlated with water-soluble organic carbon (Table 5), indicating that tomato fruit quality could be improved by adding vermicompost. Some studies have reported that tomato fruit quality can benefit from increased soil organic carbon (Jindo et al., 2016). In addition, Ca could also improve fruit quality (Kadir, 2005), and applying mixed micronutrient fertilizer (including Ca and Mg) even could reduce about 20% nitrate concentration in tomato fruit (Qin et al., 2008), which may explain that tomato in the treatment of vermicompost has relatively good fruit quality. Finally, different types of phytohormones have been found in vermicomposts (Zhang et al., 2014; Scaglia et al., 2016), and these phytohormones can significantly improve fruit quality. The use of organic fertilizer was shown to increase soil organic carbon and soil fertility, consequently resulting in a larger yield trend compared to a balanced chemical fertilizer (Gong et al., 2011). The correlations found in this study (Table 5) also imply a significant positive correlation between fruit quality, water-soluble organic carbon and soil N status.

### Effects of vermicompost on electrical conductivity in soils with different continuous growth history

The results of the present study show that vermicompost and compost had a significant impact on soil microbial C and N values (Figure S1), which are directly related to a suitable biological indicator of soil quality (Rice et al., 1996). Beneficial effects occurred even when the organic amendments were applied after 2 weeks (Figure 1), suggesting that applying organic fertilizer in a more sustainable production system could significantly improve soil fertility in just one tomato-growing period while improving the tomato yield to a level comparable to that of inorganically fertilized tomato. In particular, the long-term application of organic fertilizer can markedly improve soil quality (Jindo et al., 2016). However, due to the higher ions contained in chick manure compost (Table 2), applying compost led to higher electrical conductivity compared with vermicompost, particularly during the early growing period **(Figure 5)**, resulting in higher electrical conductivity even at the end of the growing period. Hashemimajd et al. (2004) compared vermicompost and some types of composts finding that vermicompost had the lowest electrical conductivity. Lazcano et al. (2009) even found that excessively applying composts could lead to tomato plants death due to the high concentrations of certain ions in composts and claimed that the dosage of application compost needs to be well controlled. Thus, even chick manure compost improved tomato yield and quality in this study (Figure 2), but did harm soil more than vermicompost in the way of enhancing soil electrical conductivity.

In conclusion, our results suggest that vermicompost can improve the biochemical properties of soil under different years of continuous growth, thereby increasing tomato growth, yield, and fruit quality compared with urea. Moreover, considering the higher electrical conductivity and lower soil pH achieved by applying compost, vermicompost could be a better recommendation for soils. Especially for the new soil without tomato planting history, vermicompost can produce better improvements in fruit yield and quality compared with old tomato-planting soils (Table 6). However, field studies are still needed to confirm our greenhouse results. These studies should be designed to elucidate the impacts of organic fertilizers on soil microbial processes and nutrient cycling on different soil types, to increase tomato yields under sustainable production systems. The final goal is to optimize fertilizer management to maximize yields and quality while reducing the use of inorganic fertilizer and maintaining good-quality soil.

**Table 6:** Increased or decreased percentage in fruit yield or fruit quality in vermicompost treatment compared with CK on each soil.

**Cropping years**	**Yield**	**Sugar acid ratio**	**Nitrate**	**Vc**	**Organic acids**	**Soluble sugar**
0	74 ± 12b	210 ± 42b	−31 ± 5a	47 ±1 5b	−42 ± 5b	71 ± 9b
5	43 ± 9ab	92 ± 30a	−16 ± 11a	33 ± 9ab	−36 ± 3ab	22 ± 16a
20	28 ± 4a	70 ± 9a	−16 ± 8a	18 ± 3a	−23 ± 2*a*	31 ± 5ab

“(Vermicompost – CK)/CK * 100” was used here. Means (±SE) followed by different letters denote significant differences according to Tukey test (P < 0.05) among three soil treatments under each column.

## Author contributions

X-XW analyzed the data, wrote the first manuscript and modified it. FZ performed the experiment, collected the samples and collected the data. GZ and YZ assisted to manage the experiment and collect the data. LY designed the experiments, revised the manuscript and applied funding to support the study. All the authors discussed the results and commented on the manuscript.

### Conflict of interest statement

The authors declare that the research was conducted in the absence of any commercial or financial relationships that could be construed as a potential conflict of interest.
