# Supplementary material for: Vermicompost Improves Tomato Yield and Quality and the Biochemical Properties of Soils with Different Tomato Planting History in a Greenhouse Study
Source: Front Plant Sci. 2017 Nov 21;8:1978. doi: 10.3389/fpls.2017.01978 (PMC5702354; doi:10.3389/fpls.2017.01978)
Supplement: Supplementary file 1 [file Presentation1.PDF]

Vermicompost improves tomato yield and quality and the biochemical properties of soils with different tomato planting history in a greenhouse study

Xin-Xin Wang[1, 2, §], Fengyan Zhao[1, §], Guoxian, Zhang[1], Yongyong Zhang[1] and Lijuan Yang[1, *]

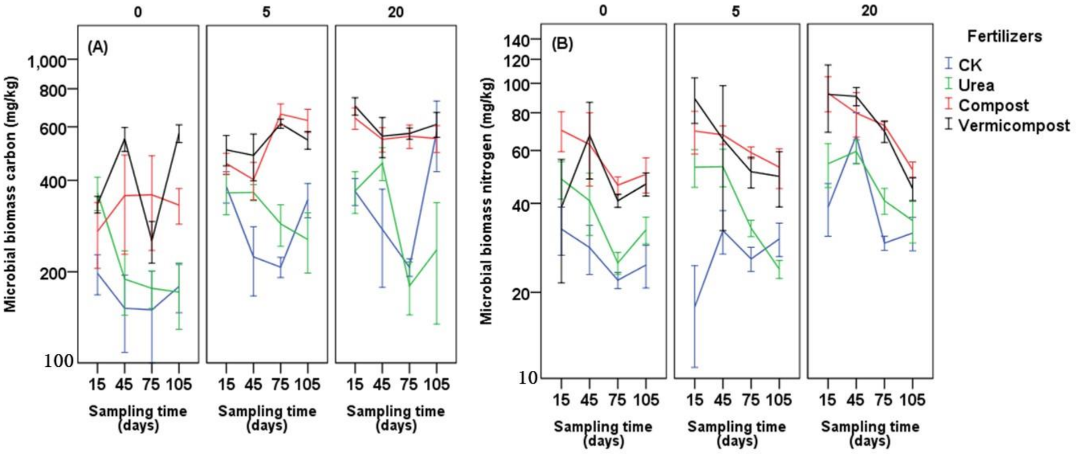

Figure S1 | Effects of fertilizers and sampling time (days after transplanting) on microbial biomass carbon (A) and microbial biomass nitrogen (B) of soils on soils with 0, 5 and 20 years of tomato planting history.
